# The State of Practice About Security in Telemedicine Systems in Chile: Exploratory Study

**DOI:** 10.2196/77395

**Published:** 2025-11-21

**Authors:** Gaston Marquez, Michelle Pacheco, Priscilla Vergara, Felix Liberona, May Chomalí, Eric Rojas

**Affiliations:** 1 Departamento de Ciencias de la Computación y Tecnologías de la Información, Universidad del Bío-Bío, Av. Andrés Bello 720, Chillán, 3780000, Chile, 56 422463324; 2 Transferencia Gestión Colaborativa en Salud Pública (Polo de Salud) para la región de Ñuble, Chillán, Chile; 3 Centro Nacional de Sistemas de Información en Salud (CENS), Santiago, Chile; 4 Institute for Biological and Medical Engineering, Pontificia Universidad Católica de Chile, Santiago, Chile; 5 Department of Clinical Laboratories, School of Medicine, Pontificia Universidad Católica de Chile, Santiago, Chile

**Keywords:** assessment, quality, security, telemedicine systems, usability

## Abstract

**Background:**

Information security within telemedicine systems is essential to advancing the digital transformation of health care. Telemedicine encompasses diverse modalities, including teleconsultation, telehealth, and remote patient monitoring, all of which depend on digital platforms, secured communication networks, and internet-connected devices. Although these systems have progressed in aligning with information security standards and regulations, there remains a shortage of comprehensive, practice-oriented studies evaluating which aspects of security are effectively addressed and which remain insufficiently managed, particularly within the Chilean context.

**Objective:**

This study aims to examine how effectively telemedicine systems in Chile address the core security attributes of confidentiality, availability, and integrity.

**Methods:**

Data were analyzed from an evaluation tool designed to assess the quality of telemedicine systems in Chile. Over a 6-year period, 25 telemedicine systems from different providers were assessed, and an in-depth examination of how companies manage key information security subcharacteristics within their systems was undertaken.

**Results:**

The findings indicate that 52% (n=13) of telemedicine systems optimally implement cryptographic techniques to protect confidentiality. In contrast, 44% (n=11) lack robust strategies for adapting to, recovering from, and mitigating security-related incidents. Fault tolerance mechanisms are frequently integrated to minimize service disruption caused by system failures. However, the prioritization of data integrity varies: while some companies treat it as a critical requirement, others assign it limited importance.

**Conclusions:**

This study offers an understanding of the security priorities and practices adopted by telemedicine providers. It highlights a prevailing tendency to prioritize security measures over usability, underscoring the need for a balanced approach that safeguards patient information while supporting efficient clinical workflows.

## Introduction

Security within telemedicine systems constitutes a critical component of the digital transformation in the health care sector [1]. The rapid expansion in the utilization of these technologies, significantly accelerated by the COVID-19 pandemic, necessitates the enhancement of mechanisms to safeguard medical information, thereby ensuring the confidentiality, integrity, and availability of patient data [2]. Telemedicine encompasses a range of modalities, including teleconsultation, telehealth, and remote patient monitoring, all of which depend on digital platforms, secured communication networks, and internet-connected devices [3]. Nevertheless, the deployment of these systems presents substantial risks, such as vulnerabilities in data transmission, unauthorized access, cybersecurity threats, and interoperability challenges among diverse health information systems. Countries generally establish guidelines for the storage and processing of personal data, which also affect telemedicine services [4]. However, despite these advancements, security in telemedicine continues to pose a global challenge due to the diversity of regulations, the rapid evolution of cyber threats, and the necessity for digital security awareness among health care professionals [5,6]. In this context, as reported by Nobili et al [7], hospitals and their information systems, including telemedicine systems, were the principal targets of cyberattacks, accounting for 42% of all computer-related incidents from 2022 to 2023.

In Chile, the regulation of personal data storage and processing is governed by law. Within the realm of telemedicine, the Chilean public health care systems have initiated efforts to enhance the digitalization of health care services among companies offering telemedicine services to their patients [8,9]. Specific guidelines have been established to safeguard patient data privacy and ensure the security of clinical information. Despite the notable advancements made by telemedicine companies in adhering to Chile’s information security guidelines and regulations, there is a paucity of systematic research that practically examines the aspects of information security that telemedicine companies effectively address and those they do not. Furthermore, there is limited information regarding the trade-offs that telemedicine companies undertake to fulfill the information security requirements of their systems.

This study presents a study examining the success levels associated with information security subcharacteristics within telemedicine companies in Chile. This study aims to identify which subcharacteristics of information security are adequately addressed and which remain insufficiently addressed by current telemedicine systems from a practical perspective. Information security was characterized through the properties of confidentiality, availability, and integrity, utilizing 7 specific subcharacteristics. The study involved an analysis of data collected from an instrument designed to assess the quality of telemedicine systems in Chile. Over a period of 6 years, the success levels of 25 telemedicine systems from different companies were evaluated, and an in-depth discussion was conducted on how these systems address the information security subcharacteristics within their telemedicine systems. The study contributes a pragmatic analysis of the priorities and actions undertaken by telemedicine companies to address subcharacteristics related to information security, as well as the trade-offs these companies encounter in ensuring security in Chile. The findings of this study offer practical insights for professionals and researchers concerned with the security of telemedicine systems.

## Methods

### Instrument

**Figure 1.**
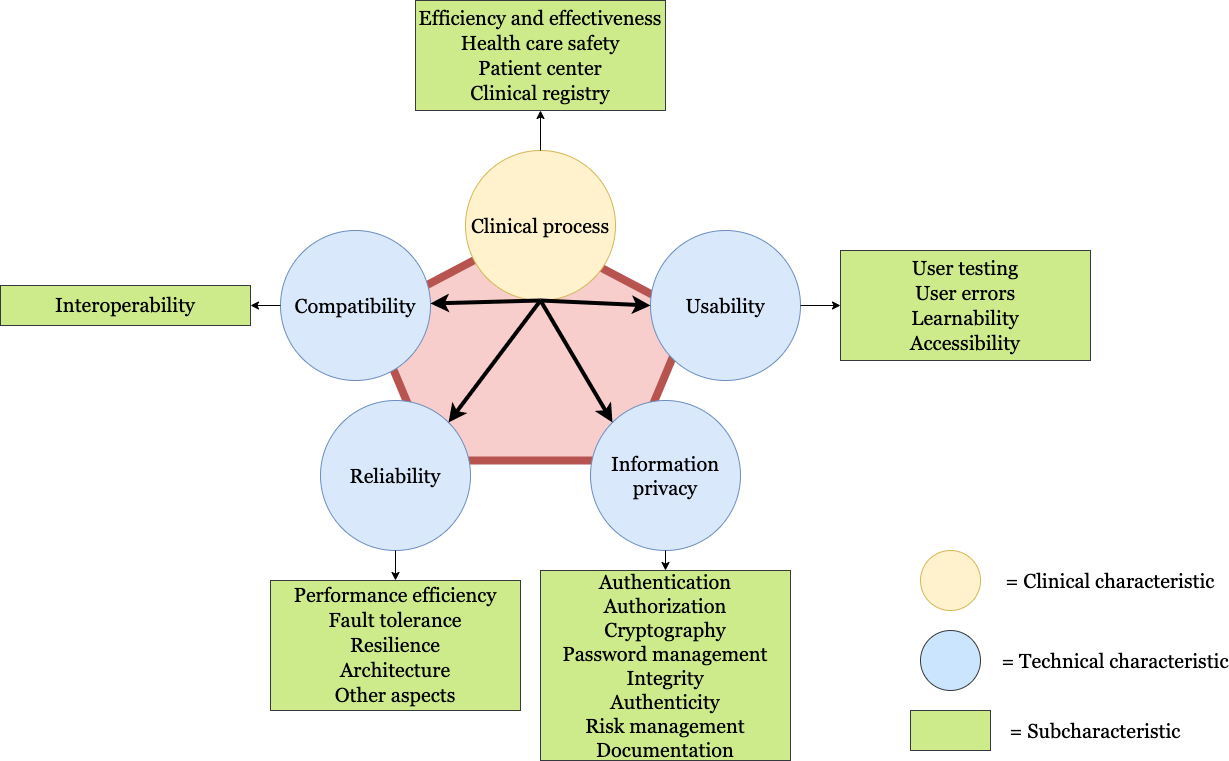
Instrument for assessing the quality of telemedicine systems, including their corresponding technical and clinical characteristics as well as their subcharacteristics.

The utilization of information technologies in health care delivery through telemedicine necessitates adherence to quality criteria typically mandated by public and private health organizations. Consequently, it is imperative to assess multiple technical and clinical aspects to ensure that the telemedicine tools employed meet the requisite minimum standards. In response to this scenario, we developed an instrument (see Multimedia Appendix 1) that evaluates the quality of telemedicine systems through an assessment process with the objective of verifying that the desired minimum requirements required by health care stakeholders are fulfilled (see Figure 1).

To validate the instrument, we conducted a comprehensive literature review to determine the elements used in the evaluation of telemedicine systems, particularly in the context of information privacy [5]. This preliminary research enabled us to identify the key security subcharacteristics considered in the assessment of telemedicine systems, as well as to derive and pinpoint additional characteristics and subcharacteristics, as illustrated in Figure 1. Subsequently, we conducted 3 pilot studies of the instrument in collaboration with (1) telemedicine experts, (2) clinical professionals, and (3) health care decision-makers. In each pilot study, we evaluated the instrument’s usefulness in assessing Chilean public telemedicine systems. At the conclusion of each pilot, we collected feedback from participants and revised the instrument accordingly. Thereafter, we engaged 3 telemedicine companies, partners of our research group, to apply the revised instrument to their telemedicine systems. Upon the completion of the system evaluations, we considered the observations made by each company’s teams and further refined the instrument.

The evaluation process focuses on 2 characteristics: clinical and technical. Each characteristic evaluates multiple aspects. In the clinical characteristic, the context, description of the telemedicine solution, and role of the patient in the clinical process are evaluated. For this reason, we evaluated the following subcharacteristics: (1) efficiency and effectiveness, (2) health care safety, (3) patient center, and (4) clinical registry. These subcharacteristics represent the most relevant concerns regarding telemedicine in Chile. In the technical characteristic, 4 properties are evaluated: compatibility, usability, reliability, and information privacy. In turn, each characteristic has 18 subcharacteristics representing different concerns. The decision to evaluate the clinical and technical dimensions is based on the priorities faced by health decision-makers in Chile. These priorities aim to address laws that have been enacted in the country, which are oriented toward cybersecurity, data privacy, interoperability, and patient protection.

Each subcharacteristic evaluates the telemedicine company’s adherence to both technical and legal standards. Consequently, the instrument requires the telemedicine company to provide pertinent information and documentation from the telemedicine system to confirm the accuracy of the responses recorded in the instrument. Given the complexity of evaluating telemedicine systems, our instrument employs a simplified scale for stakeholder comprehension. The scale considers a score of 3 points for optimal, 2 points for acceptable, 1 point for risky, and 0 points for unsatisfied.

An optimal score is defined when the telemedicine company proposes novel mechanisms or methodologies that exceed the baseline expectations defined by the instrument in each subcharacteristic; that is, when the telemedicine company implements additional or more advanced security techniques to provide a higher degree of patient trust. Some of these techniques include hash encryption algorithms, role and access management through identifiers, zero trust architecture, intrusion detection, multifactor authentication, and network micro-segmentation, among others. Similarly, an acceptable score is defined when the telemedicine company provides the explicit evidence required by the instrument and complies with the legal regulations associated with each subcharacteristic. A company receives a risk score when it does not explicitly describe the required evidence and does not fully comply with the legal regulations associated with each subcharacteristic. Finally, an unsuccessful score corresponds to a company that neither provides evidence nor complies with regulations.

### Research Objectives

In this study, we focused on examining subcharacteristics related to security. Therefore, the conceptualization of security is structured into 3 fundamental properties: confidentiality, availability, and integrity. Confidentiality pertains to the protection of information, ensuring that access is restricted to authorized individuals by preventing unauthorized access, improper disclosure, or data breaches [10]. Availability guarantees that information is accessible and usable when needed by authorized users [11]. Integrity ensures that information remains accurate and reliable, without unauthorized modifications [11].

Correspondingly, as elucidated by the instrument presented in Figure 1, we established that the confidentiality characteristic encompasses 4 subcharacteristics: authentication, authorization, cryptography, and authenticity. Authentication refers to the process of verifying the identity of a user, system, or device prior to granting access to a resource or service [12]. Authorization involves determining the actions or resources that an authenticated user is permitted to access [12]. Cryptography is the study of techniques for safeguarding information by converting data into an encrypted format, which can only be read or modified by authorized individuals or systems [13]. Information authenticity ensures that data originate from a legitimate source and have not been altered during transmission or storage [12].

Concurrently, the availability characteristic is composed of 2 subcharacteristics: fault tolerance and resilience. Fault tolerance refers to a system’s capacity to maintain proper functionality despite the failure of 1 or more of its components [14]. Resilience, on the other hand, denotes a system’s ability to adapt, recover, and continue operations following an adverse event, such as a cyberattack, hardware malfunction, or natural disaster [15].

Therefore, we define the following objectives:

1. Identify security-related subcharacteristics that telemedicine companies successfully address. This objective is oriented toward analyzing the subcharacteristics of the instrument that perform optimally in the context of security, demonstrating telemedicine companies’ ability to meet information security requirements.
2. Investigate the factors enabling telemedicine companies to achieve optimal and acceptable scores in security-related subcharacteristics. This objective aims to explore the key elements that contribute to successful performance in the security subcharacteristics.
3. Determine the security-related subcharacteristics that telemedicine companies fail to effectively address. This objective focuses on identifying subcharacteristics that exhibit low scores and understanding their implications for information security.
4. Analyze the reasons telemedicine companies fail to achieve favorable scores in security-related subcharacteristics. This objective aims to uncover the underlying challenges or limitations that prevent companies from meeting their security requirements.
5. Examine the trade-offs telemedicine companies create between usability and security in their systems. Usability and security frequently exhibit conflicting objectives [11]. While usability aims to simplify access and enhance user experience, security focuses on safeguarding data and systems, which typically necessitates the implementation of restrictions or additional measures to ensure such protection. Therefore, this objective is oriented toward understanding how usability considerations influence security decisions and how companies balance these 2 critical aspects within their systems.

Although there are regulations and security standards, such as the Health Insurance Portability and Accountability Act (HIPAA) [16], ISO/IEC 27001 [17], Control Objectives for Information and Related Technologies [18], and the NIST Cybersecurity Framework [19], not all can be fully applied to the reality of telemedicine in Chile owing to work culture, institutional organization, and the level of maturity of telemedicine systems. For this reason, our instrument draws on these regulations and standards to adapt security assessments to the Chilean context, with the aim of establishing an initial security baseline for telemedicine systems in Chile.

### Research Questions

We defined the following research question:

RQ1: Which subcharacteristics of telemedicine systems address security concerns? Rationale: The objective of this research question is to elucidate the subcharacteristics of our instrument that performs optimally in the context of security. While the instrument employs a multidimensional approach, this research question aims to demonstrate the capacity of telemedicine companies to meet information security requirements.

RQ2: Which subcharacteristics of telemedicine systems do not address security concerns? Rationale: In contrast to RQ1, this research question aims to identify the subcharacteristics that exhibit low scores within the security context. In addition, this research question is intended to describe the reasons telemedicine companies are unable to achieve favorable security scores.

RQ3: In the context of usability, which trade-offs do telemedicine companies make to ensure security within their systems? Rationale: Addressing security necessitates a balance between the properties and quality of telemedicine systems. This research question aims to elucidate the trade-offs that our instrument identifies when addressing information security in telemedicine systems.

### Data Collection

**Figure 2.**
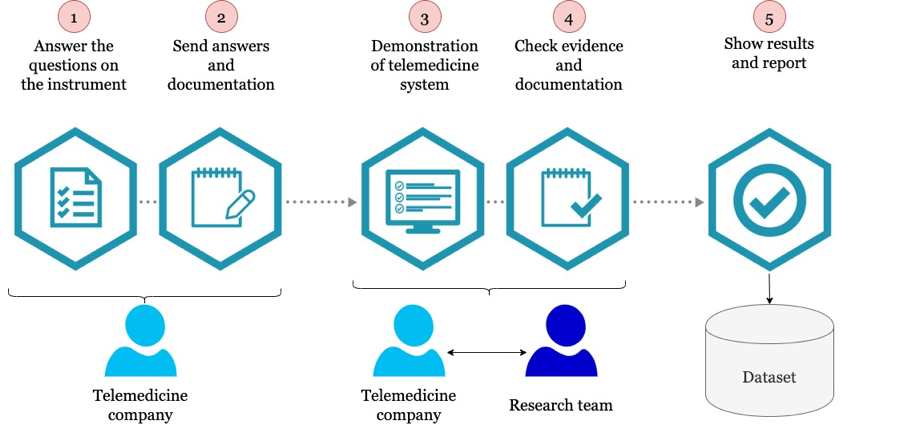
Process for collecting data from telemedicine companies as well as from their telemedicine systems.

The data collection process is illustrated in Figure 2.

The data collection process is characterized by 2 distinct perspectives. First, it involves a self-assessment process conducted by the telemedicine company, wherein the company’s technical and clinical representatives utilize our instrument to respond to key inquiries and provide corresponding documentation to substantiate their responses. Second, our research team performs a cross-validation of the documentation and responses obtained from the instrument through a demonstration of the telemedicine system, focusing on corroborating the answers provided in the instrument. The selection of the 25 telemedicine companies was based on a technical analysis of the telemedicine system each company offered, the number of clients, and the volume of telemedicine services provided to both public and private health care services in Chile. In addition, the quality agreements held by the companies were reviewed, as these had been granted by public health institutions, given that this sector serves the largest number of patients in Chile. We used a nonprobabilistic, convenience-based sampling approach. Specifically, we engaged companies willing to participate in order to provide them with reports on the security and quality of their systems, thereby supporting the continuous improvement of their security mechanisms.

In the initial phase, the telemedicine company is tasked with assembling clinical and technical experts to address all inquiries associated with our instrument. The instrument was developed within a web-based system that facilitates the upload of documentation in various formats. Upon the compilation of all information and the completion of the instrument’s inquiries by the telemedicine company, the subsequent phase involves transmitting the information to our research team. Upon receiving notification from the company and verifying that no information was pending, we informed the telemedicine company of the assessment’s initiation. The third phase aims to mitigate subjectivism and bias in the company’s responses by demonstrating the functionalities associated with the inquiries evaluated by the instrument. For this phase, our research team convenes with the stakeholders of the telemedicine company to discuss pertinent aspects of the instrument. Subsequently, in the fourth phase, the research team organizes to evaluate the telemedicine company’s documentation and demonstration to assign a score to each subcharacteristic that our instrument assesses. The assessment is conducted by 3 members of the research team and subsequently validated by a senior researcher. The 3 investigators independently evaluated the responses and documentation submitted by the telemedicine company, ultimately determining the final score through consensus. Following score assignment, a preliminary report is generated, which must be approved or rejected by a senior researcher on the team. The company initiates an improvement process in which the aspects to be improved are addressed until the necessary score for approval is reached. The number of interactions depends on how the company presents corresponding evidence and documentation. If the report is approved, the fifth phase consists of creating a formal instance to present the results of our analysis and inform the company of its qualification. Additionally, the entire analysis and its results are stored in a dataset utilized for research purposes.

### Data Analysis

We used descriptive statistics and manual analysis of documentation to address the research questions. About RQ1 and RQ2, we analyzed the frequency of scores to categorize the number of companies that achieved optimal, acceptable, risky, and unsuccessful scores.

Regarding RQ1 and RQ2, we conducted a comprehensive review of all documents and software artifacts that our instrument mandates companies to submit for the evaluation of their telemedicine systems, in addition to responding to the questions within the instrument. For each assessed security subcharacteristic, companies are required to furnish evidence demonstrating their compliance with the subcharacteristic, thereby substantiating the responses provided in the instrument. In our study, we requested explicit evidence from companies to ensure the objectivity of the evaluation. For instance, when companies submit evidence of authentication, they typically provide documentation on the authentication mechanisms implemented in the telemedicine system, along with source code packages. This procedure is similarly applied to other security subcharacteristics. Finally, a team of researchers independently assessed the evidence and provided a review, which was subsequently verified by a senior researcher on the team.

Concerning RQ3, the subcharacteristics related to security are classified into 2 categories: successful and not successful. The subcharacteristics categorized as “successful” are those that obtained optimal (score 3) and acceptable (score 2) scores. Conversely, subcharacteristics categorized as “not successful” are those that received risky (score 1) and unsatisfied (score 0) scores. Subsequently, we compared the percentages of usability and security. The percentage of successful corresponds to the ratio of subcharacteristics that are categorized as “successful” compared to the total (subcharacteristics with “successful” and “not successful” categories).

### Ethical Considerations

According to the applicable ethical and legal framework in Chile, including Law No. 19.628 on the Protection of Private Life, research ethics review is required for studies involving human participants, biological samples, or personal data [20]. As this study did not involve the collection, processing, or analysis of any personal, sensitive, or health-related data, it was not subject to review by an institutional ethical research board.

## Results

In relation to RQ1, Table 1 shows that telemedicine systems prioritize cryptography as principal subcharacteristic to ensure security.

A total of 13 companies, representing 52% of the total, obtained the optimal score as they considered cryptographic methods to be pertinent to ensuring the protection of sensitive patient data as well as the security of patient-physician communication. With the exception of the subcharacteristic of authenticity, where the distribution of scores is more equitable, in the subcharacteristics of authorization and fault tolerance, the acceptable score leads to the preferences (15 companies representing 60% are inclined toward this score in these 2 subcharacteristics). In the same context, authentication and integrity also share the same preferences for this score (10 companies representing 40% favor these 2 subcharacteristics). Table 2 summarizes the main reasons identified in the documentation provided by the companies for which telemedicine companies achieve favorable scores in security-related subcharacteristics.

**Table 1.** Frequency of scores obtained by telemedicine companies.

		Subcharacteristic	Optimal	Acceptable	Risky	Unsatisfied
Security	Confidentiality	Authentication	3	10	10	2
		Authorization	6	15	2	2
		Cryptography	13	8	1	3
		Authenticity	7	8	7	3
	Availability	Fault tolerance	6	15	3	1
		Resilience	4	7	3	11
	Integrity	Integrity	2	10	10	3

**Table 2.** Summary of the main reasons telemedicine companies achieve optimal and acceptable scores.

Subcharacteristic	Reason
Cryptography	Most telemedicine systems use AWS Cognito, SHA^a^ 256, or HTTPS^b^ as cryptographic tools. This means that the data encryption acts over the TLS^c^. In addition, most telemedicine systems have their infrastructure hosted in the cloud, mainly in AWS^d^. All telemedicine systems use cryptographic mechanisms for video encryption. DTLS^e^ 1.2 or SRTP^f^ is the most commonly used method for encrypting transmitted data.
Authentication	Most telemedicine systems employ username and password authentication. Furthermore, these systems utilize 2-factor access to enhance security mechanisms. Nevertheless, we observed that although the instrument requires explicit information regarding access to sensitive data, not all companies provide evidence of role-based access control within their telemedicine systems.
Authorization	Telemedicine companies have access control mechanisms that use specific permission profiles for each type of user. Nevertheless, not all companies explicitly describe whether they have validation from the Chilean Registry of Individual Health Care Providers to access data.
Authenticity	Telemedicine systems use unique and random login identifiers. This identifier changes when the user is authenticated. However, we note that some companies do not describe evidence that a single sign-on is implemented in the telemedicine system.
Fault tolerance	Most telemedicine systems have implemented functionalities that offer patients the possibility of resuming consultations in case of failure through a link and code provided by email. Although companies are able to identify failure scenarios and implement prevention and mitigation measures, not all companies are able to guarantee response times to allow users to continue using the services in case of failure.
Integrity	Telemedicine companies manage sensitive data through security protocols that classify information into public, proprietary, client, and privileged categories. They recognize the confidentiality of patients’ clinical information, applying stringent protection measures.

a SHA: Secure Hash Algorithm.

b HTTPS: Hypertext Transfer Protocol Secure.

c TLS: Transport Layer Security.

d AWS: Amazon Web Services.

e DTLS: Datagram Transport Layer Security.

f SRTP: Secure Real-time Transport Protocol.

Regarding RQ2, Table 3 describes that the subcharacteristic with the lowest score corresponds to resilience. A total of 11 companies (44% of the total number of companies analyzed) did not address methodologies to adapt, recover, and overcome security-related situations effectively. As in RQ1, authentication and integrity exhibit equivalent frequencies of scores, but in this instance with respect to the risk score.

**Figure 3.**
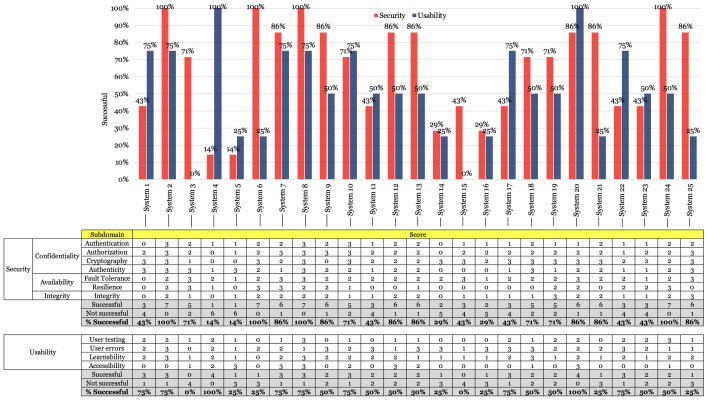
Distribution of percentages of success with security and usability.

Regarding RQ3, Figure 3 compares the results of the security and usability subcharacteristics with their corresponding successful-related percentages for each company. The results reveal variations in success levels, ranging from significant to subtle.

For system 1, the system satisfied 75% of the usability subcharacteristics. However, in terms of security, the system only satisfies 43% of the subcharacteristics, with all availability and integrity subcharacteristics remaining unaddressed. For systems 2 and 3, both address a greater proportion of security subcharacteristics compared to usability subcharacteristics, with system 2 addressing 100% of the subcharacteristics. However, system 3 demonstrated inadequate performance in cryptography, despite exhibiting a higher successful percentage in security. System 4 focuses exclusively on usability but only successfully addresses 1 security subcharacteristic. It is noteworthy that this company does not address fundamental security standards such as cryptography and authorization.

Although system 5 demonstrates no significant difference between the successful-related percentages for usability and security, the overall percentage remains low. This indicates that the company maintains equilibrium between both properties, albeit at a substantially low level of success. Systems 6-9 have successfully addressed security concerns, but with varying levels of usability success: company 6 addressed only 25% of usability criteria, while systems 7-9 exceeded 50%.

**Table 3.** Summary of the main reasons telemedicine companies achieve risky and unsuccessful scores.

Subcharacteristic	Reason
Resilience	Documentation submitted by telemedicine companies indicates that they use a resilient mechanism in their telemedicine systems. However, when analyzing the documentation submitted to the instrument, companies do not necessarily explicitly describe a recovery plan.
Authentication	Table 2 indicates that the companies do not provide precise information regarding their roles. In these instances, companies attempt to elucidate role-based authentication mechanisms but omit pertinent information. However, certain companies, despite mentioning the utilization of roles for authentication, merely attach screenshots or provide no information whatsoever.
Integrity	Companies often lack explicit methods for eliminating unnecessary data and inadequately address error detection in data entry within telemedicine systems.

Systems 10 and 11 exhibit a situation analogous to system 4, wherein they maintain a balance in addressing security and usability. However, system 10 demonstrates higher successful-related percentages compared to system 11. Systems 12 and 13 yielded identical results, significantly favoring security measures. System 14 maintains equilibrium in satisfying usability and security requirements, albeit at notably low percentages. In this regard, the company focuses exclusively on cryptography and fault tolerance as security mechanisms. Concerning usability, they have successfully addressed only user error control measures.

System 15 prioritized security over usability; however, it exhibited a notably low successful percentage. Similar to system 14, system 16 achieved equivalent percentages of success in both usability and security. System 17 placed greater emphasis on usability compared to security, specifically focusing on user error control in terms of usability. Systems 18 and 19 demonstrated identical results regarding success with security versus usability. Nevertheless, system 18 made a concerted effort to develop a user-accessible system.

System 20 distinguishes itself through high successful levels in both security and usability. In this regard, the company surpassed others in terms of usability and security success. System 21 addresses security to a greater extent than usability. However, this company does not delineate any process to address the resilience of telemedicine systems. System 22 satisfied more usability subcharacteristics than security subcharacteristics. System 23 demonstrates a balanced, albeit low, successful percentage. Systems 24 and 25 exhibited a trend of addressing more security subcharacteristics than usability subcharacteristics.

## Discussion

### Overview of the Findings

The instrument proposed in this study delineates the state of practice of characteristics that define the structure of telemedicine systems. The objective of this study is to elucidate the state of security from 3 perspectives: confidentiality, availability, and integrity.

From a confidentiality perspective, the results elucidate significant findings for the analysis. Regarding authentication, organizations balance acceptable and risky outcomes in the authentication mechanisms implemented in their telemedicine systems. As authentication verifies the identity of the user attempting access by validating credentials, 10 companies achieve acceptable results, while 10 companies exhibit risky outcomes. Among the acceptable results, organizations have adopted mechanisms that extend beyond basic username and password management. For instance, these companies utilize multifactor authentication to provide an additional security layer. Conversely, companies with risk scores limit themselves to the minimum requirements in user and password management. Analyzing the extreme scores, 3 companies achieved optimal results by not only implementing multifactor authentication but also incorporating biometric authentication features for users. Regarding unsuccessful scores, these companies demonstrated no evidence of documentation pertaining to authentication.

From the perspective of authorization, 15 companies adhere to acceptable mechanisms for authorizing access to sensitive data. It is deemed acceptable for a company to not only utilize basic authorization mechanisms but also to adapt to governmental regulations regarding access to clinical data. In Chile, specifically, there are legally mandated regulations governing access to sensitive information. In total, 4 companies that received risky or unsuccessful scores did not fully comply with these regulations. Conversely, 6 companies achieved an optimal score in authorization due to their implementation of internal processes and protocols for data authorization. This scenario is advantageous for security as it systematizes activities related to authorization, thereby facilitating traceability and auditing.

Cryptography is the preferred subcharacteristic for telemedicine companies. It was observed that 13 companies focused their efforts on providing adequate levels of cryptography using tools or algorithms specifically designed for this purpose. Similarly, 8 companies implemented cryptographic levels using libraries from development frameworks (eg, OAuth) but did not extend their efforts beyond those companies with an optimal score. Conversely, 1 company received a risk score because although it claimed to use cryptographic mechanisms, it provided no supporting evidence. Finally, 3 companies failed to meet cryptographic standards. This deficiency is primarily attributed to a lack of knowledge among telemedicine system developers regarding the implementation of cryptographic methods in their systems.

With regard to authenticity, ensuring that data, messages, or transactions originate from legitimate and reliable sources and have not been altered during transmission or storage is a primary concern for telemedicine companies. Although 8 companies adequately met the fundamental requirements for authenticity, 7 companies distinguished themselves by implementing nonrepudiation methods in their authentication processes. Specifically, these companies ensure that the data cannot be modified and that the sender cannot deny having generated the data. However, 7 companies failed to adequately describe the authenticity mechanisms in their systems. Furthermore, it was observed that some companies conflated the concept of authenticity with networking.

About availability, the results demonstrated disparities between fault tolerance and resilience. In terms of fault tolerance, 15 companies with acceptable scores implemented cluster strategies and load balancing. These companies opted for these techniques due to the availability of various technology stacks in the market that support their implementation. Furthermore, 6 companies employed additional mechanisms related to fault tolerance, specifically failover mechanisms. This technique ensures that in the event of a component or service failure, the system automatically transitions to an alternative resource capable of performing the same function. Conversely, it is imperative to note that the results presented in Table 1 elucidate a substantial disparity between resilience mechanisms.

A total of 11 companies failed to delineate the methodologies employed in their systems to address resilience, and 3 companies provided ambiguous indications of resilience, lacking sufficient clarity. The research team found that explicit references to resilience were uncommon among telemedicine companies, suggesting that further understanding of the related techniques and technologies could be beneficial. Among the companies that address resilience, 7 adhere to the recommendations from the Chilean Ministry of Health concerning the application of resilience in health information systems. In total, 4 companies implemented additional techniques specifically related to server redundancy.

Regarding integrity, the results in Table 1 are analogous to those of the authentication. The highest scores are concentrated in the acceptable and risky categories. The 10 companies that achieve an acceptable score primarily focus their efforts on delineating processes and mechanisms involving encryption protocols, such as Transport Layer Security [21], to safeguard data from tampering during transmission. Furthermore, 2 companies distinguished themselves in integrity by implementing hashing algorithms to verify data integrity.

**Figure 4.**
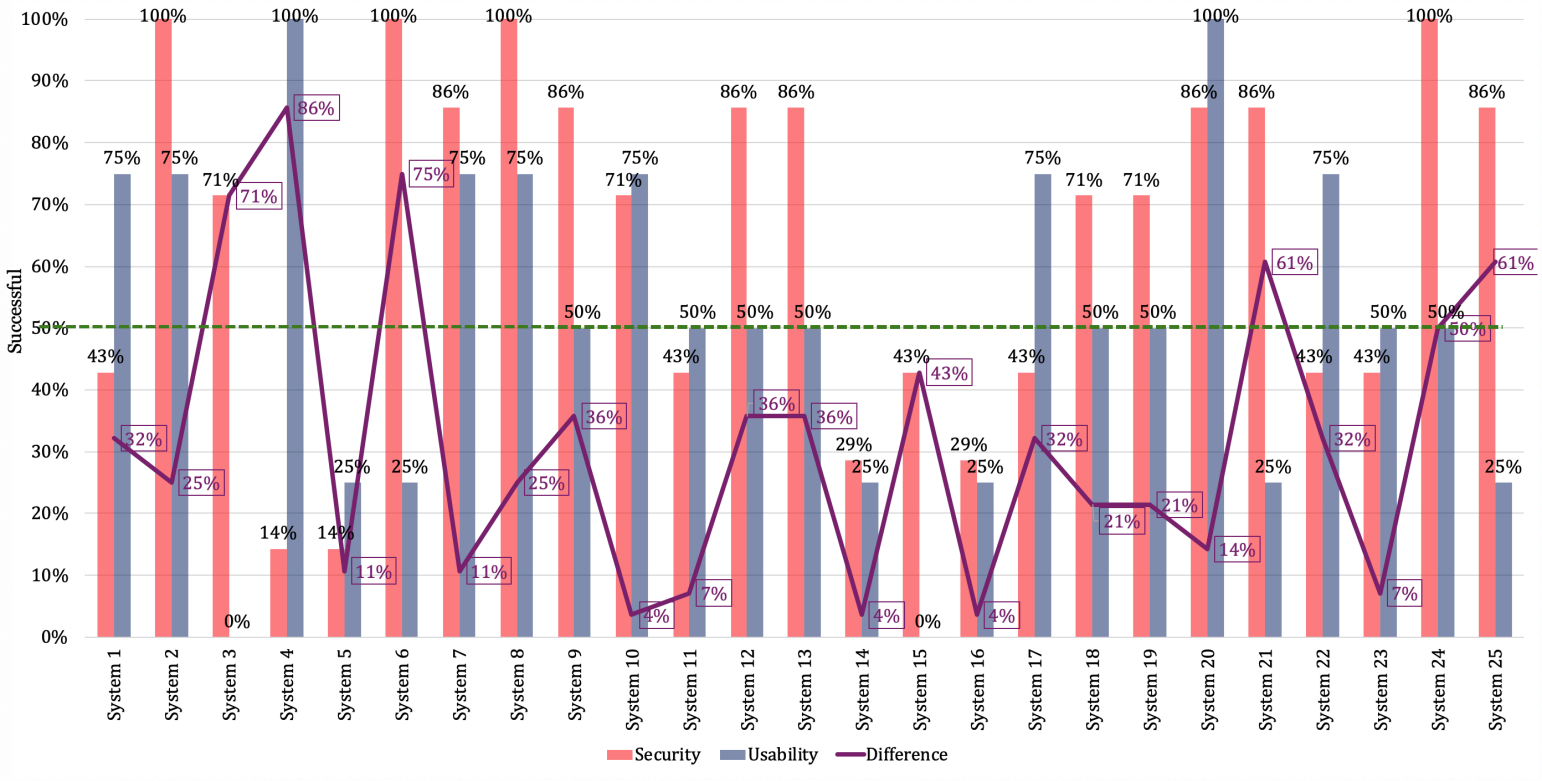
Distribution of the difference in percentages of success concerning security and usability. The dashed line represents the upper and lower 50% threshold.

Conversely, 10 companies attain risky results as they inadequately meet the minimum requirements for information integrity. In this regard, these companies have made insufficient efforts to address data integrity in teleconsultations. Figure 4 describes that telemedicine companies generally prioritize security over usability, although the disparity in successful-related percentages exhibits volatility.

Utilizing 50% as a threshold to differentiate successful levels, certain companies, specifically companies 3, 4, 6, 20, 21, and 25, described a difference in percentages exceeding 50%. In these instances, the trends are distinct, favoring either usability or security. Conversely, in the remaining companies, the difference between the percentages was less than 50%. While this indicator does not comprehensively reflect the actual situation of companies, it serves as a parameter to determine the nature of trade-offs they make. Among the 6 companies with a difference greater than 50%, 5 exhibited a tendency to prioritize security in their telemedicine systems over usability, while 1 company demonstrated an inclination in the opposite direction.

### Principal Results

Cryptography is the subcharacteristic in which we observed the highest proportion of organizations that attained the maximum score. This phenomenon may be attributed to the prevalent perception that cryptography constitutes the most efficacious and widely utilized method for data protection and security. Of the 25 companies evaluated, 13 achieved an optimal score in cryptography, while 8 companies attained an acceptable score.

Another aspect in which the organizations demonstrated positive performance is fault tolerance. Although the majority of organizations performed adequately in this characteristic, most did not attain the highest scores. This can be attributed to limitations in infrastructure, scalability, dependence on third-party services, and integration with other systems. Most organizations are likely capable of managing basic faults, such as implementing backup mechanisms or addressing minor interruptions, but lack robust solutions to mitigate major failures such as complete system crashes, loss of critical data, or failures during high-demand situations.

In contrast, a significant negative aspect is recovery capability. The suboptimal results in this characteristic can be attributed to various factors, one of which is successful fault-tolerance scores. Both aspects are closely related as they address how systems manage failures. The recovery capability focuses on how the system restores itself after failure. Many organizations may prioritize developing fault-tolerant systems while neglecting the implementation of postfailure recovery measures. This situation can be problematic because although fault tolerance results are generally positive, most organizations did not achieve the highest scores, suggesting that their systems may not be resilient to severe errors. By not prioritizing recovery capability, a significant failure could render it extremely challenging to restore the system to its normal state.

Another subcharacteristic in which the majority of companies demonstrated suboptimal results is accessibility. The inadequacy of accessible systems impedes the utilization of these platforms by individuals with special needs as well as the elderly population, who frequently experience visual or auditory impairments. This deficiency represents a missed commercial opportunity, as a substantial number of elderly individuals and persons with disabilities could potentially derive significant benefits from telemedicine. Due to the absence of adequate accessibility options, these companies have restricted access to a crucial demographic segment, resulting in biased systems that can only be effectively utilized by young and middle-aged adults.

The findings identified critical areas with direct practical implications for the development of telemedicine systems. The high scores in cryptography indicate a strong emphasis on data protection; however, organizations must continue updating these measures to counter evolving threats. The moderate level of fault tolerance suggests basic resilience but also highlights vulnerabilities during significant system failures, necessitating investment in scalable and robust infrastructure. The poor performance in recovery capability is particularly concerning, as it may result in extended service downtime and compromise patient safety, underscoring the need for formal disaster recovery plans.

### Limitations

First, the assessment of security in telemedicine systems frequently presents numerous challenges due to the complex and sensitive nature of clinical data and the system itself. Consequently, the development of an instrument to evaluate the current state of a telemedicine system across various characteristics may inadvertently omit aspects that are fundamental to telemedicine. From a systemic perspective, system evaluation encompasses diverse complexities and details, ranging from requirement identification to software architecture. Nevertheless, international regulations such as the HIPAA [16], the European Union’s General Data Protection Regulation [22], and the Fast Healthcare Interoperability Resources or Health Level Seven standard [23], among others, establish various levels and concerns that must be addressed by telemedicine systems. Furthermore, the policies and regulations of individual countries constitute perspectives that must be considered in any instrument designed to evaluate telemedicine systems.

Second, the instrument proposed in this study to evaluate telemedicine systems has limitations in encompassing all variants and scenarios that should be considered in telemedicine. It is conceivable that this instrument may require adaptation to the diverse regulatory frameworks for telemedicine across different countries. Nevertheless, this instrument represents the first endeavor inspired by international telemedicine regulations and is focused on the Chilean context. This initial approach has been refined and endorsed by public and foreign health decision-makers in Chile, resulting in the participation of 25 companies in this study to determine the preliminary status of their telemedicine systems’ quality. Regarding security, the results of this study enable the determination of the current status of fundamental aspects of confidentiality and privacy in telemedicine systems. While a comprehensive security assessment should consider multiple levels of abstraction, such as infrastructure, networks, and providers, this study represents an initial step in the pragmatic discussion of security in telemedicine systems within the Chilean context.

Third, the instrument described in this study does not evaluate the security techniques and mechanisms employed by telemedicine companies. Specifically, the instrument is not designed to empirically analyze cryptographic techniques or compare software architecture styles, components, or technological infrastructure. The scale assessed the level of evidence provided by the company regarding the implementation of security subcharacteristics in telemedicine systems. A more comprehensive and empirical analysis of the quality and efficacy of the techniques, processes, mechanisms, and methods utilized by telemedicine companies to implement security-related subcharacteristics should be conducted in a separate study. Additionally, in our study, we primarily focused on identifying the security mechanisms employed by telemedicine systems, rather than conducting an in-depth analysis of which techniques are more significant than others. This level of detail would require examining the source code of the systems and applying more advanced comparative methods to determine the degree of importance. Therefore, our intention was to illustrate to the reader what telemedicine systems are currently implementing in terms of security, rather than analyzing the complexity of the security mechanisms themselves.

Fourth, the 6-year span of our study may not fully capture the evolution of security threats related to telemedicine systems. The field of information security and cybersecurity in telemedicine is continuously evolving in terms of vulnerability techniques and methods. In this context, our study focuses on the responses provided by telemedicine system developers, without delving into whether the techniques implemented in these systems remain adequate in light of emerging security scenarios. Nevertheless, Chilean regulations require telemedicine companies to demonstrate, through a series of processes and software artifacts, that they comply with governmental information-security standards. Consequently, companies providing telemedicine services are obliged to stay up to date with new types of security incidents. Therefore, during the 6 years covered by the study, the information provided by the telemedicine companies at least meets Chilean regulations on information security in telemedicine.

While the limitations outlined in this section may affect the generalizability of our study’s findings, the instrument we developed is adaptable to a range of contexts and security environments. At present, the instrument is tailored to the Chilean context. Nevertheless, as telemedicine regulations in Chile are largely based on international standards, the instrument can be adapted to suit different scenarios in other countries. Furthermore, although adverse security scenarios in telemedicine continue to evolve, our instrument enables a cross-sectional analysis of security, which may subsequently inform more in-depth analyses of each subcharacteristic. In this regard, our study focuses on the evidence provided by telemedicine companies concerning how they address the various aspects of security; however, it does not consider how these companies adapt to different security scenarios.

### Future Work

Future research will concentrate on the systematic refinement and validation of the assessment instrument employed in this study, ensuring its alignment with the evolving landscape of telemedicine technologies and emerging regulatory requirements. While the current version offers a practical evaluation of key dimensions, such as security, interoperability, and usability, the rapid advancement of digital health tools, particularly those incorporating artificial intelligence, introduces new challenges in risk management, data governance, and ethical compliance.

In the Chilean context, recent legislative developments concerning cybersecurity, personal data protection, and artificial intelligence–driven clinical decision support systems underscore the urgency of equipping telemedicine providers with tools that not only evaluate but also anticipate compliance gaps and technological vulnerabilities. This calls for a research agenda oriented toward the codesign of adaptive, evidence-based instruments that integrate technical, clinical, and legal perspectives, thereby supporting secure and more trustworthy digital health services.

### Comparison With Prior Work

Poleto et al [24] addressed the increasing adoption of telemedicine systems in Brazil, driven by the necessity for health care access in remote areas and expedited implementation during the COVID-19 pandemic. The authors proposed the utilization of fuzzy cognitive maps to analyze the complexity of cybersecurity, translating expert knowledge into maps that represent causal relationships between security concepts, facilitating scenario creation, and enhancing cybersecurity strategies. For security assessment, 15 variables influencing cyberattacks were identified and validated by an information technology manager, and a fuzzy cognitive map diagram was constructed. This study demonstrates how changes and incorrect configurations can overburden servers and how insufficient investment can generate inefficiencies, emphasizing the significance of controlling information technology services.

Vidanagamachchi et al [25] conducted an analysis of the privacy and security challenges in telemedicine systems in Sri Lanka, emphasizing their significance due to the rapid adoption precipitated by the COVID-19 pandemic. Despite technological advancements and the implementation of government policies for digitalization, deficiencies in patient data protection persist due to the lack of specificity in the regulations. The authors propose a self-assessment mechanism for patients to evaluate the security of telemedicine apps. To this end, a questionnaire was developed encompassing 7 critical characteristics related to telemedicine and cybersecurity, considering the demographic and ethnic diversity of Sri Lanka. The evaluation involved collecting responses from 100 users utilizing an online questionnaire. The results indicated that the majority of participants do not peruse privacy and security policies and are unaware of storage capabilities and backup policies.

Poleto et al [26] emphasized the significance of managing cyber risks in telemedicine, particularly in the exchange of medical images, due to the susceptibility of systems to cyberattacks. They proposed a cybersecurity risk management framework for telemedicine utilizing a bowtie analysis approach that integrates fault tree analysis and event tree analysis. To assess security, the framework identifies risk factors and potential risk events, prioritizes actions to mitigate the impact of cyberattacks, and provides recommendations for establishing effective security policies. This framework is predicated on the ISO/TS 13131:2014 standard, which delineates recommendations for developing quality objectives and guidelines for telehealth services. The study also elucidates the importance of control measures to ensure the confidentiality, integrity, and availability of medical information.

Zhou et al [27] addressed concerns regarding privacy and security in telemedicine services within the context of increased utilization driven by the necessity to enhance health care access in rural areas. They proposed the development and validation of a self-assessment questionnaire for telemedicine providers to evaluate their privacy and security measures. This questionnaire was constructed based on a systematic review of security practices and the HIPAA audit protocol. The study encompassed 31 telemedicine providers who responded to 49 questions in the questionnaire. The results demonstrated that the questionnaire exhibits high reliability for assessing privacy and security practices in telemedicine systems and identified key vulnerabilities in areas such as data storage, secure networks, encryption, data backup, and informed consent.

Kim et al [1] examined the necessity of managing security risks in telemedicine systems due to the increased utilization of these technologies for remote diagnosis and prescription, particularly in the context of infectious diseases such as COVID-19, SARS-CoV, and MERS-CoV. This investigation proposes the development of a security risk assessment model for telemedicine systems utilizing an attack tree approach with attack occurrence probability and attack success probability as variables. Security threats were analyzed across 7 characteristics of telemedicine services using data collected through surveys conducted at a medical institution. The model was implemented to identify system vulnerabilities.

The studies analyzed offer diverse perspectives on security within telemedicine systems and the approaches to addressing these concerns. Several of these perspectives are reflected in the security subcharacteristics examined in our study, as they are broadly applicable to telemedicine systems. Furthermore, these studies align with the cross-sectional analysis of security policies and practices in telemedicine systems in Chile, allowing us to adapt our study to different national contexts. However, most existing studies rely primarily on theoretical frameworks, high-level risk assessment models, or self-assessment questionnaires that provide general guidance but lack direct application to operational telemedicine environments. In contrast, our study adopts a practice-oriented approach that examines how security is effectively implemented and perceived in real telemedicine systems used in Chile. This approach contributes to a contextualized understanding of how security measures are operationalized in Chile with evolving telehealth regulation, thereby complementing and extending the insights offered by previous international frameworks.

### Conclusions

This study presents an investigation into the security status of telemedicine systems. Utilizing an instrument developed by the research team, the study analyzed 25 telemedicine companies and their respective systems based on 3 critical security attributes: confidentiality, availability, and integrity. The analysis aims to identify the subcharacteristics of confidentiality, availability, and integrity that telemedicine companies effectively address and the underlying reasons for their success. Conversely, the study also aims to identify the primary reasons for telemedicine companies’ failure to adequately address security and the factors contributing to these shortcomings. Furthermore, the study explores the trade-offs involved in ensuring security in telemedicine, particularly concerning the usability of telemedicine systems.

We investigated 3 research questions relating to the security subcharacteristics addressed by telemedicine systems (RQ1), those that are not addressed (RQ2), and the trade-offs telemedicine companies make to ensure security (RQ3). With regard to RQ1, the findings revealed that cryptography is the principal subcharacteristic of interest in telemedicine systems in relation to confidentiality. In terms of availability, fault tolerance is consistently addressed to prevent disruptions in medical care caused by system failures. Data integrity emerges as a variable concern among telemedicine providers; some consider it highly important, while others do not regard integrity as a particularly relevant characteristic. Concerning RQ2, the subcharacteristic receiving the least attention is resilience. The findings for RQ3 indicated that telemedicine companies generally prioritize security over usability.

The findings of this study highlight critical areas with direct practical implications for the advancement of telemedicine systems. Strong performance in cryptography reflects a clear commitment to secure data; however, organizations must ensure these measures are continuously updated to address emerging cyber threats. The moderate level of fault tolerance indicates some degree of system resilience but also exposes vulnerabilities during major failures, emphasizing the need for investment in scalable and robust infrastructure. Of particular concern is the weak performance in recovery capability, which could lead to prolonged service interruptions.

## Supplementary material

10.2196/77395 Multimedia Appendix 1 Instrument used in our research and its corresponding questions by characteristic.

## Abbreviations

HIPAA: Health Insurance Portability and Accountability Act
